# Effect of *Bacillus subtilis* isolated from yaks on D-galactose-induced oxidative stress and hepatic damage in mice

**DOI:** 10.3389/fmicb.2025.1550556

**Published:** 2025-03-05

**Authors:** Lei Wang, Aoyun Li, Xiaohu Zhang, Mudassar Iqbal, Zain Ul Aabdin, Mengen Xu, Quan Mo, Jiakui Li

**Affiliations:** ^1^College of Veterinary Medicine, Huazhong Agricultural University, Wuhan, China; ^2^College of Veterinary Medicine, Henan Agricultural University, Zhengzhou, China; ^3^Department of Preventive Veterinary Medicine and Public Health, Faculty of Veterinary and Animal Sciences, Ziauddin University, Karachi, Pakistan; ^4^College of Animal Science, Xizang Agricultural and Animal Husbandry University, Nyingchi, China

**Keywords:** yak, *Bacillus subtilis*, oxidative stress, hepatic injury, Keap1/Nrf2 signaling pathway, gut microbiota

## Abstract

Acute hepatic injury is a severe condition that is always accompanied by oxidative stress and inflammation, seriously threatening the health of the host. Probiotics have been shown to be involved in the regulation of antioxidant system and gut microbiota activity, but studies on the effects of yak derived *Bacillus subtilis* (*B. subtilis*) on acute liver injury and oxidative stress remain scarce. Here, we aim to explore the ameliorative effects of *B. subtilis* isolated from yaks on oxidative stress and hepatic injury caused by D-galactose, as well as the underlying processes. Results indicated that *B. subtilis* administration, particularly the BS3, significantly mitigated hepatic damage induced by D-galactose in mice as evidenced by ameliorating liver tissue damage as well as decreasing ALT (*p* < 0.05) and AST (*p* < 0.05) levels. Additionally, the *B. subtilis* intervention was demonstrated to enhance the antioxidant system in D-galactose-exposed mice, as manifested by increased T-AOC and SOD, alongside a decrease in MDA levels (*p* < 0.05). Meanwhile, *B. subtilis* intervention could effectively mitigate oxidative damage via modulating the Keap1/Nrf2 signaling pathway. Importantly, *B. subtilis* exhibited a pronounced protective effect against D-galactose-induced intestinal barrier dysfunction through improving tight junction proteins. The gut microbiota results suggest that BS3 alters the abundance of some gut flora such as Firmicutes phylum and *Oscillibacter* and *Lachnospiraceae_NK4A136* genera, which affects the composition of the gut microbiota and reverses the decrease in the microbial richness index in mice. In summary, these findings demonstrated that *B. subtilis* isolated from yaks serve as a promising candidate to ameliorate oxidative damage and hepatic injury. Meanwhile, the positive regulation effect of *B. subtilis* on gut microbiota and intestinal mucosal barrier may be one of its underlying mechanisms to alleviate oxidative stress and hepatic injury.

## Introduction

Oxidative stress has been increasingly implicated in a wide range of diseases, driving progressive damage and functional impairment in crucial organs, such as the liver (Kimball et al., 2021). Previous research has identified that inadequate clearance of accumulated reactive oxygen species by intracellular antioxidant defense mechanisms is main cause of oxidative stress (Panda et al., 2022). In general, both enzymatic and non-enzymatic antioxidant systems play crucial roles in mitigating oxidative stress and maintain homeostasis (Zhou et al., 2018). However, consumption of high-fat meals, excessive alcohol intake, smoking, and exposure to environmental toxins consistently result in the production and accumulation of a significant amount of oxidants (Yang et al., 2020; Paithankar et al., 2021). As oxidants accumulate, they readily extract electrons from interacting molecules, including cellular macromolecules. This process sets off a cascade of reactions that ultimately cause damage to cell structure, leading to a variety of chronic diseases that are closely linked to alterations in the gut microbiota (Filomeni et al., 2015; Wang et al., 2018).

The gastrointestinal system, as the primary organ for nutrient absorption and digestion, harbors over 2,000 distinct microbial species (Loomba et al., 2017; Li et al., 2021; Zhou et al., 2023). The importance of gut microbiota to immunity, metabolism, and nutritional absorption has been shown (Wang B. et al., 2021; Wang C. et al., 2021). Additionally, the gut microbiota contributes to sustain the gut barrier’s integrity, which is necessary for protecting gut homeostasis and averting pathogen invasion (Yang J. et al., 2021). The gut microbiota is a crucial biotransformer that transforms nutrients into metabolites, e.g., indole derivatives and other short chain fatty acids (SCFAs) that are vital to gut homeostasis (Rodriguez et al., 2020). Nevertheless, gut microbiota is highly susceptible to external factors, with oxidative stress being a particularly disruptive influence (Wu et al., 2021; Wu Y. et al., 2022).

The liver plays a crucial role as a central organ in metabolism and detoxification in both human and animal physiology, making it especially susceptible to oxidants and various exogenous stimuli (Prieto and Monsalve, 2017). Its functionality is intricately linked to the gastrointestinal tract via the portal venous system, where gut microbiota and its metabolites are critical to maintaining hepatic health (Wang J. et al., 2022). Reciprocally, the liver facilitates intestinal homeostasis by secreting bile acids and antimicrobial peptides into the gastrointestinal tract, which aids in the regulation of microbial proliferation and contributes to the maintenance of gut microbial balance (Chi et al., 2019). Disruption of the bidirectional link between the liver and the gut can precipitate a range of diseases, e.g., hepatitis and cirrhosis (Wan et al., 2022). Numerous researches show that imbalances in gut microbiota disrupt intestinal function, fostering the selective proliferation of pathogenic bacteria. This imbalance can lead to metabolic dysregulation, sub-health and even severe diseases (Nishida et al., 2018; Franzosa et al., 2019). In cases of gut microbial dysbiosis, an abundance of harmful microbes and their metabolites may be transferred to the liver, thus exacerbating liver damage and disease progression (Wang G. et al., 2021). Therefore, an effective strategy to mitigate oxidative stress-related liver disease is to maintain the gut microbial balance.

Probiotics are beneficial commensal bacteria in the gut that confer multiple advantages to the host, including antimicrobial activity and immunity regulation (Kong et al., 2021). Probiotics are able to attenuate oxidative damage by regulating gut microbiota and metabolism. In Xu’s et al. (2019) research, they reported that *Lactobacillus* supplementation effectively reduced LPS-induced oxidative injury, Huang et al. (2019) demonstrated the significant efficacy of *L. plantarum C88* in preventing aflatoxin B(1)-induced liver injury. While supplementation with lactic acid bacteria has shown promise in reducing oxidative stress and liver-related diseases (Xu et al., 2019), *Bacillus subtilis* is also a commonly used probiotic that preserves appropriate niches for microorganisms, enhances nutrient utilization, and inhibits pathogens (Rhayat et al., 2017; Ghimire et al., 2024). Extensive studies have demonstrated that *Bacillus subtilis* has a positive effect on growth performance, health status, and gut morphology in hosts (Fan et al., 2013; Manafi et al., 2017). However, little is known about the effects of *B. subtilis* on oxidative stress and related liver damage. The yak is a valuable and rare plateau breed, concentrated mainly on the Tibetan plateau at altitudes above 4,000 meters. It is widely acknowledged that yaks have extremely strong heart and lung functions, considerable amounts of red blood cells, and a high haemoglobin content, which allows them to obtain sufficient oxygen at high altitudes. Thus, yaks are referred to as the “boat of the plateau” as they can endure the harsh environment, extreme altitudes, low oxygen levels, and oxidative stress. These superior characteristics of the yak are most likely related to probiotics in the gut. In the research, our goal is to investigate the effect of *B. subtilis* isolated from yaks against oxidative stress and hepatic injury caused by D-galactose in mice as well as the underlying processes.

## Materials and methods

### Animal experiment and sample acquisition

The *B. subtilis* strains were isolated from healthy yaks (aged 1 year) living in the Tibetan plateau at an altitude of over 4,000 meters. In addition, isolated strains of *B. subtilis*, including BS3 and BS7, were preserved at the Animal Nutrition & Metabolism Disorders Research Center of Huazhong Agricultural University.

One hundred and five healthy Kunming mice (weighing 40 ± 5 g) were obtained from the laboratory animal centre of Huazhong Agricultural University. These mice were placed in the recommended conditions of 23 ± 2°C temperature, 55 ± 5% humidity, normal light–dark cycle and free drinking water. All the rats were fed the commercial standard diet (Liang Liang, Shenyang). These mice underwent a acclimatization period of 7 days in their rearing environment to minimize stress and then randomly assigned to seven groups (*n* = 15 per group), i.e., (1) the control group (C group): 0.2 mL normal saline (NS) by gastric gavage followed by intraperitoneal injection of NS (0.2 mL) daily for 5 weeks, (2) the D-galactose-induced model group (DG group) was established according to Xu’s et al. (2019) research, receiving NS (0.2 mL) via gastric gavage prior to the intraperitoneal injection of D-galactose (0.2 mL) daily for 5 weeks, (3) the vitamin C treatment group (VC group): vitamin C (0.2 mL) was infused via a gastric tube daily before intraperitoneal D-galactose (0.2 mL) for 5 weeks, (4) the *B. subtilis* 3 (BS3) supplement group (BSA group): 0.2 mL BS3 (10^8^ CFU/mL) by gastric gavage prior to NS (0.2 mL) via intraperitoneal injection daily for 5 weeks, (5) the *B. subtilis* 7 (BS7) supplement group (BSB group): 0.2 mL BS7 (10^8^ CFU/mL) by gastric gavage prior to NS (0.2 mL) via intraperitoneal injection daily for 5 weeks, (6) the BS3 intervention group (TBSA group): 0.2 mL BS3 by gastric gavage prior to D-galactose (0.2 mL) via intraperitoneal injection daily for 5 weeks, (7) the BS7 intervention group (TBSB group): 0.2 mL BS7 by gastric gavage prior to D-galactose (0.2 mL) via intraperitoneal injection daily for 5 weeks. The entire experimental period lasted for 6 weeks (1 week acclimatization period +5 week experiment time) during which the mice were weighed weekly.

On day 42, prior to euthanasia, blood was obtained from the tail vein of the mice using a vacuum and a sterile needle (5 gauge), and the liver, kidney, spleen, colon, and colonic contents were collected. Thereafter, these organs were carefully weighed, and the respective organ indices (for kidney, spleen, and liver) were calculated according to the formula: visceral index (%) = organ wet weight (g)/body weight (g) × 100. A portion of the liver (about 1–1.5 cm^3^) and colon (about 2 cm) were fixed in 4% paraformaldehyde solution with a view to subsequent pathological observation, while the remaining tissues and contents were stored at −80°C for molecular biological experiments.

### Analysis of serum biochemistry and antioxidant capacity

Blood samples were centrifuged for 20 min (3,000 rpm/min) and serum was extracted by inhaling supernatant. Serum biochemical indices such as alanine aminotransferase (ALT) and aspartate aminotransferase (AST) were quantified using the automatic biochemical analyzer (Mairui, Shenzhen). Concurrently, the antioxidant capacity was assessed using commercial ELISA assay kits for total antioxidant capacity (T-AOC), glutathione peroxidase (GSH-Px), catalase (CAT), superoxide dismutase (SOD). Additionally, the oxidative damage index malondialdehyde (MDA) was also detected (Jiancheng, Nanjing).

### Histological observations

Fixed tissues, including the liver and colon, are flattened, dehydrated, and cleared by ethanol and xylene. The dehydrated tissues were subsequently encased in paraffin and sliced into 4 μm thick sections. H&E (haematoxylin and eosin) staining was performed in accordance with our previous work (Wang et al., 2024), including dewaxing of paraffin sections, H&E staining, and section sealing after dehydration. Finally, tissue sections were observed under an inverted microscope (BH-2, OLYMPUS).

### Western blotting

Proteins from colon and liver samples of all groups were extracted and quantified using RIPA Lysis Buffer (Beyotime, Shanghai) and BCA protein kit (Beyotime, Shanghai), respectively. Subsequently, 10 μL of proteins at the same concentration (5 mg/mL) were electrophoresed on 5–20% SDS-PAGE gels and transferred to the PVDF membranes (0.22 μm), which were then incubated with primary antibodies against occludin (1:1,000), claudin-1 (1:1,000), β-actin (1:1,000), KEAP1 (1:1,000), NRF2 (1:1,000), HO-1 (1:1,000) and NQO1 (1:1,000) (ABclone, China) for 16 h. Subsequently, membranes were washed with TBST for 30 min, followed by incubated with the secondary antibody (ABclone, China) for 2 h. After further washing with TBST for 30 min, protein bands were visualised using an ECL reagent (Beyotime, Shanghai). Finally, the protein bands of the image were analyzed by ImageJ software.

### 16S rRNA sequencing analysis

Six samples per group were used for 16S rRNA sequencing. Bacterial genomic DNA of colon contents was extracted by using the QIAamp DNA Mini Kit. The extracted DNA was assessed by 0.8% agarose gel electrophoresis. Primers targeting bacterial 16S rDNA (338F: ACTCCTACGGGGAGGCAGCA and 806R: GGACTACH VGGGTWTCTAAT) were designed to amplify the V3/V4 region, and PCR amplification was performed based on previous research (Wang et al., 2024). The PCR products were detected by 2% agarose gel electrophoresis, and target fragments were recovered via an AXYGEN gel recovery kit. Following preliminary quantification results from electrophoresis, PCR amplification and recovery products were quantified using fluorescence. After PCR amplification, the resulting high-quality products are utilized for the construction of sequencing libraries, which are subsequently combined and sequenced using a MiSeq platform.

### Sequencing data analysis

The raw data undergoes rigorous initial quality screening to ensure the reliability of subsequent bioinformatics analyses. This process included trimming primer sequences, removing chimeras, and eliminating short or mismatched sequences. Subsequently, validated sequences with 97% similarity were clustered and partitioned into OTUs. The QIIME software was used for the calculation of the alpha analysis indices. Additionally, beta analysis was conducted and visualized through principal coordinate analysis (PCoA) to determine the alternation of gut microbial profile between groups. The specific microbial composition was analyzed at phylum and genus levels, respectively. Significant differences in taxa were determined using LEfSe and Metastats analyses.

### Statistical analysis

The experimental data were analyzed using SPSS software (Version 23), and the results were presented as mean ± SD. GraphPad Prism (Version 9) was utilized for generating figures. The symbol # indicates comparison with the C group, while * represents comparison with the D-galactose induced oxidative injury group (DG group). In this context, #*p* < 0.05 or *p < 0.05 indicates that the difference is statistically significant.

## Results

### Growth performance

**Figure 1:**
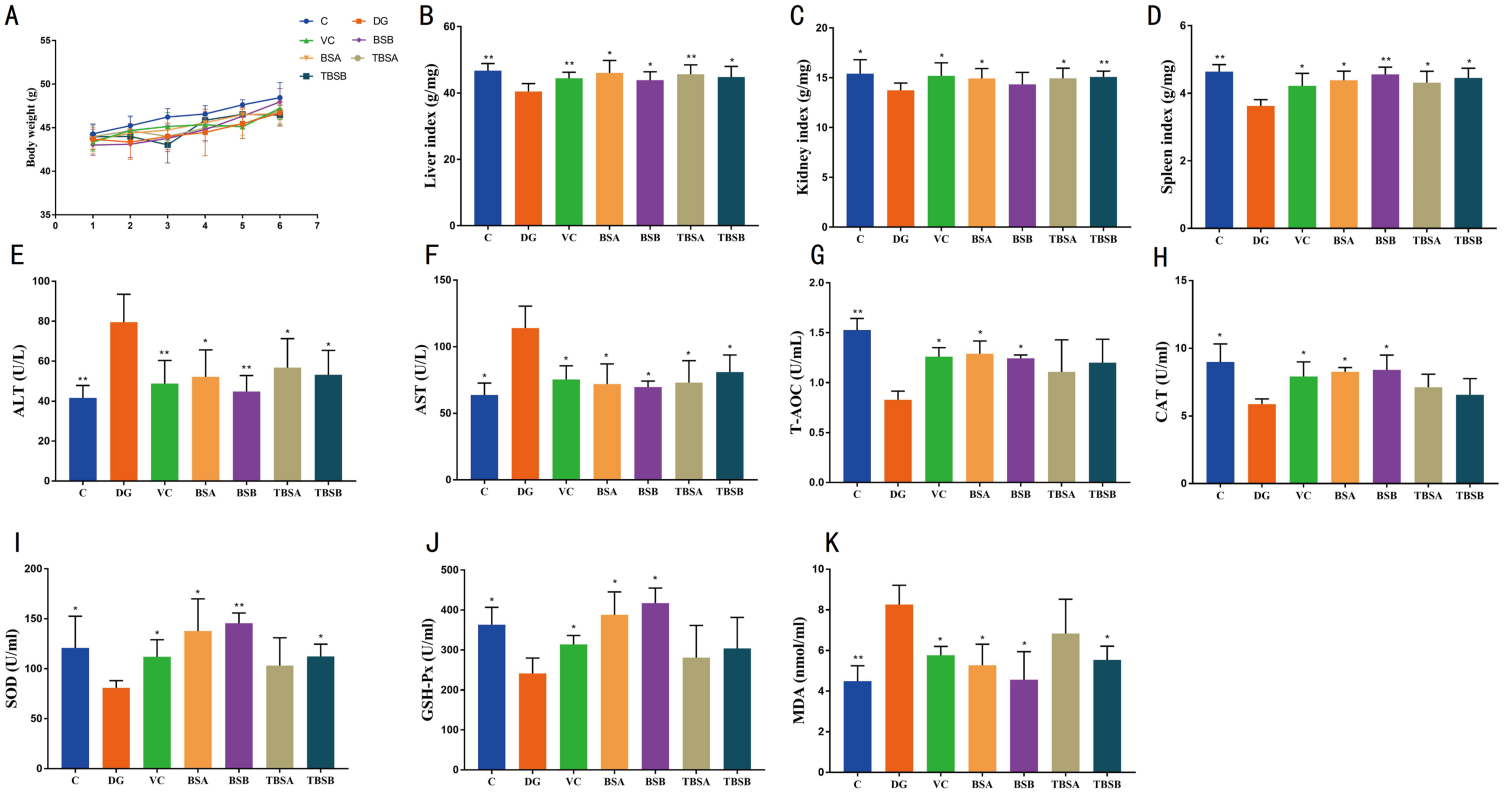
The intervention of *B. subtilis* significantly alleviated the negative effects induced by D-galactose. **(A)** Changes in body weight across seven groups. The organ indices for the liver, kidney, and spleen are depicted in **B–D**, respectively. **(E,F)** Represent the serum concentrations of ALT and AST, respectively. The serum antioxidant capacity, as illustrated in **G–K**, encompasses the levels of T-AOC, CAT, SOD, GSH-Px, and MDA. C: the control group, DG: the D-galactose-induced model group, VC: vitamin C treatment group, BSA: the BS3 supplement group, BSB: BS7 supplement group, TBSA: the BS3 intervention group (i.e., BS3 treatment + D-galactose), TBSB: the BS7 intervention group (i.e., BS7 treatment + D-galactose). The symbol # indicates comparison with the C group, while * represents comparison with the D-galactose induced oxidative injury group (DG group).

There was no significant difference in body weight between groups (Figure 1A), but D-galactose (DG) treatment resulted in a significant decrease in liver (*p* < 0.01), kidney (*p* < 0.05) and spleen indices (*p* < 0.01) in mice (Figures 1B–D). However, interventions with *B. subtilis* (BS3 and BS7) and VC restored visceral indices (i.e., liver, kidney and spleen indices) to the level of the C group, as evidenced by the absence of significant differences between groups C, VC, TBSA (D-galactose + BS3) and TBSB (D-galactose + BS7). It is noteworthy that supplementation with BS3 or BS7 alone had no effect on visceral indices and body weight in mice.

### Biochemistry and antioxidant analysis

To evaluate the potential of *B. subtilis* in alleviating hepatic oxidative damage induced by D-galactose, the serum levels of ALT and AST were assessed (Figures 1E,F). The findings revealed a significant increase in ALT and AST levels in the DG group compared to the C group (ALT: *p* < 0.01, AST: *p* < 0.05). Conversely, interventions with BS3, BS7, and VC effectively reduced the levels of ALT and AST (*p* < 0.01 or *p* < 0.05), restoring them to normal levels similar to those of the C group. Notably, compared to the C group, supplementation with BS3 or BS7 alone had no effect on ALT and AST levels.

Additionally, the changes in the antioxidant indices among the groups were analyzed. The results showed significantly higher MDA levels in the DG group than the C group (*p* < 0.01), along with lower CAT (*p* < 0.05), T-AOC (*p* < 0.01), SOD (*p* < 0.05) and GSH-Px (*p* < 0.05) levels compared to the C group, indicating that D-galactose expose significantly decreased the antioxidant capacity of the mice. However, the treatments of D-galactose + BS3, D-galactose + BS7, and D-galactose + VC, respectively, increased T-AOC and SOD while decreasing MDA indices (*p* < 0.05) compared to the D-galactose exposed group. Moreover, these groups including VC, TBSA, and TBSB exhibited no significant difference compared to the C group. Therefore, intervention with BS3, BS7 as well as VC appeared to mitigate this oxidation damages caused by D-galactose exposure (Figures 1G–K).

### Histological observations

**Figure 2:**
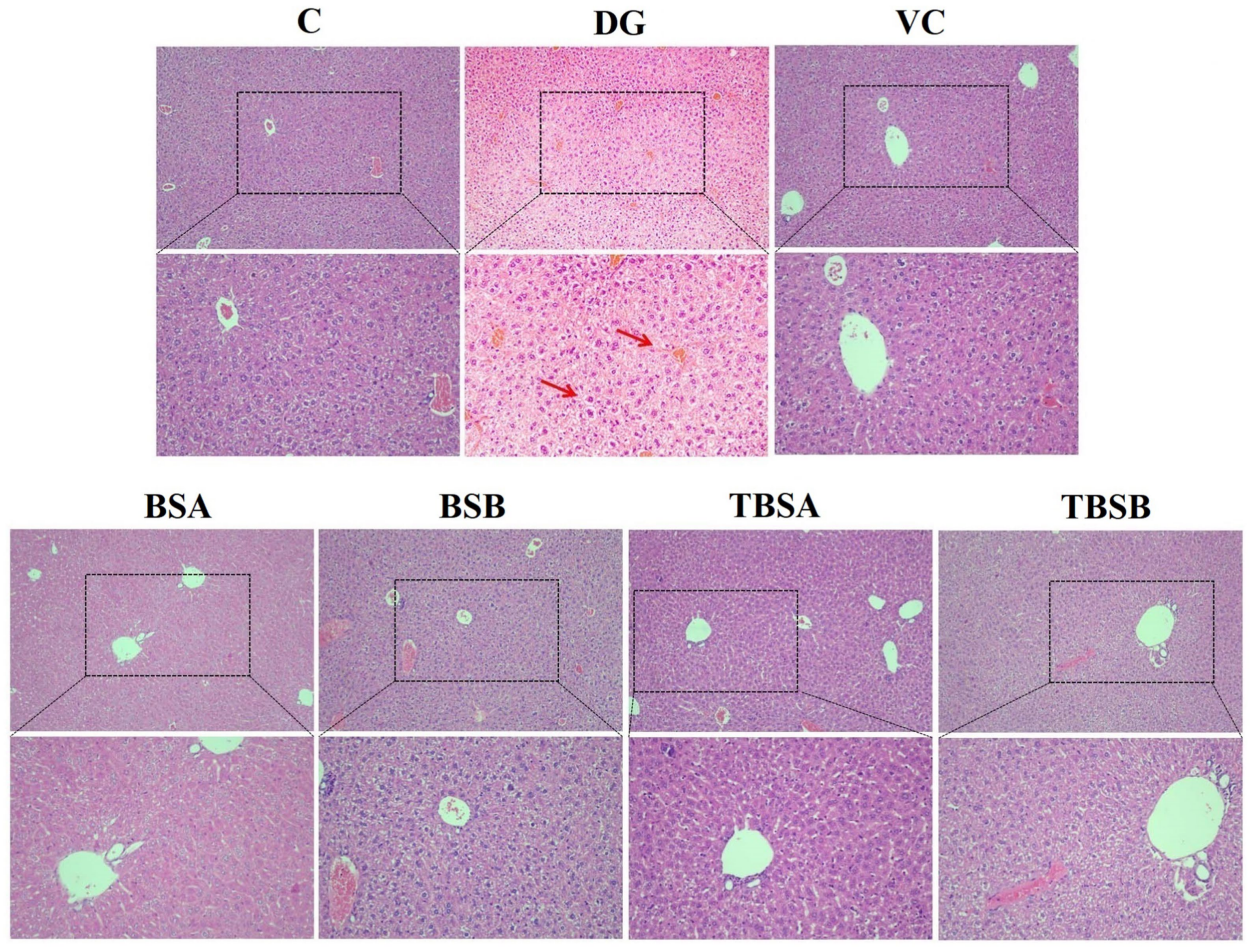
The histological morphology of the liver in groups C, VC, BSA, and BSB demonstrated a regular and healthy structure, while the DG group exhibited evident pathological changes, such as the red arrow indicating vacuolar degeneration. In contrast, the TBSA and TBSB group showed a significant alleviation of vacuolar degeneration. The scale bars are 100 μm and 50 μm, respectively.

**Figure 3:**
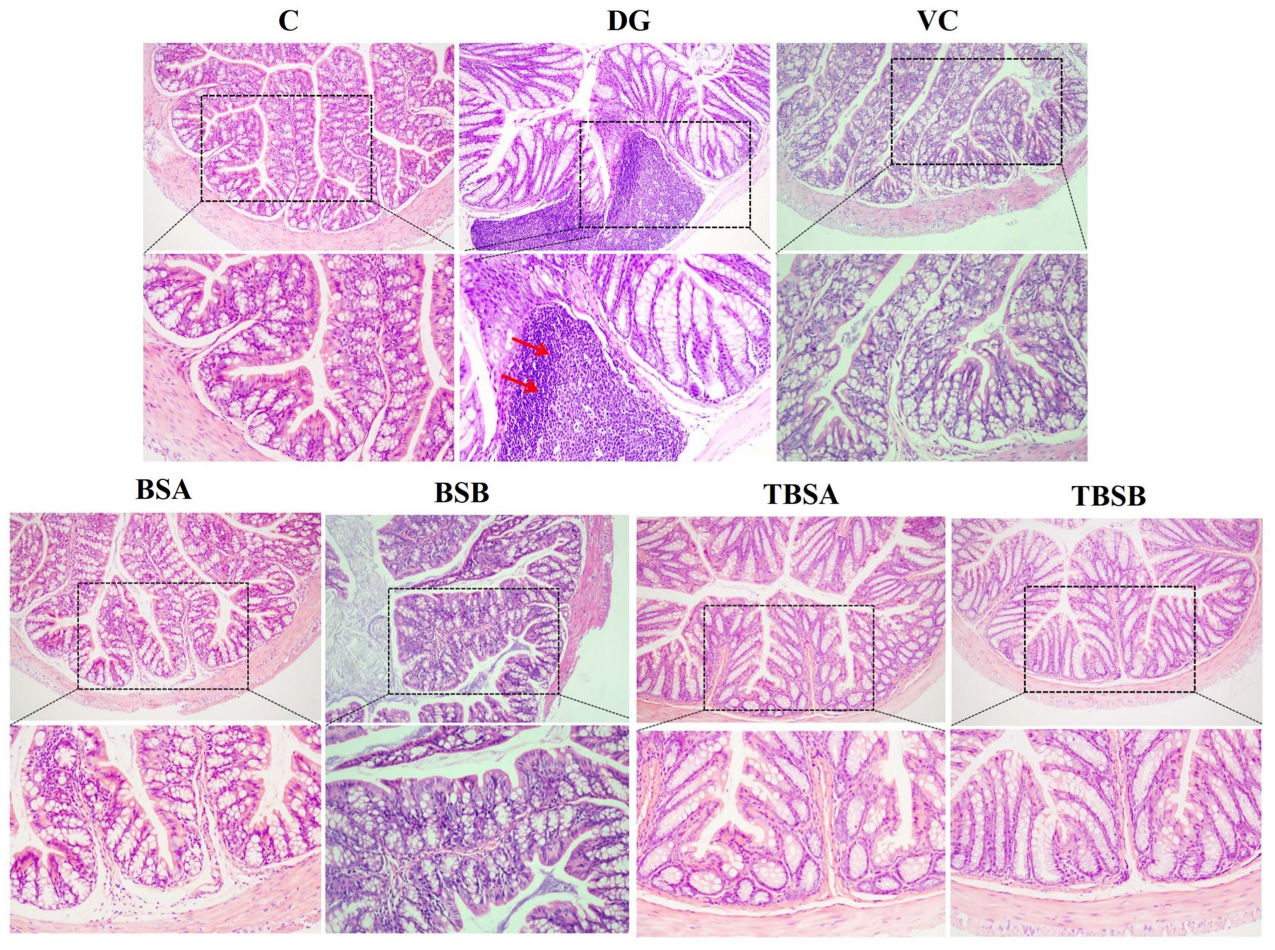
The HE staining of colon tissue in different groups. The colon structure of groups C, BSA, and BSB appeared normal and healthy, without any obvious pathological changes. In contrast, the DG group exhibited significant pathological changes, such as the red arrow indicating inflammatory cell infiltration. However, the intervention of VC (VC), BS3 (TBSA), and BS7 (TBSB) resulted in a notable decrease in inflammatory cell infiltration. Scale bars: 100 μm or 50 μm.

Histopathological analysis, as shown in Figures 2, 3, revealed that the liver structure in groups C, BSA and BSB appeared normal and healthy with no obvious pathological changes. Conversely, D-galactose exposure (DG group) resulted in significant liver vacuolar degeneration. Notably, interventions with BS3, BS7, and Vitamin C (VC) significantly alleviated vacuolar degeneration in the liver relative to the DG group (Figure 2).

Pathological alterations were also observed in colonic tissue. D-galactose caused significant inflammatory cell infiltration in the colon. However, treatment with BS3, BS7 and VC interventions significantly alleviated inflammatory cell infiltration. Additionally, supplementation with BS3 and BS7 alone did not induce any pathological changes and showed no obvious differences compared to the C group (Figure 3). Thus, it can be concluded that BS3, BS7, and VC were safe and effective treatments for colonic inflammation induced by D-galactose.

### *Bacillus subtilis* mitigate D-galactose-induced gut barrier damage in mice

**Figure 4:**
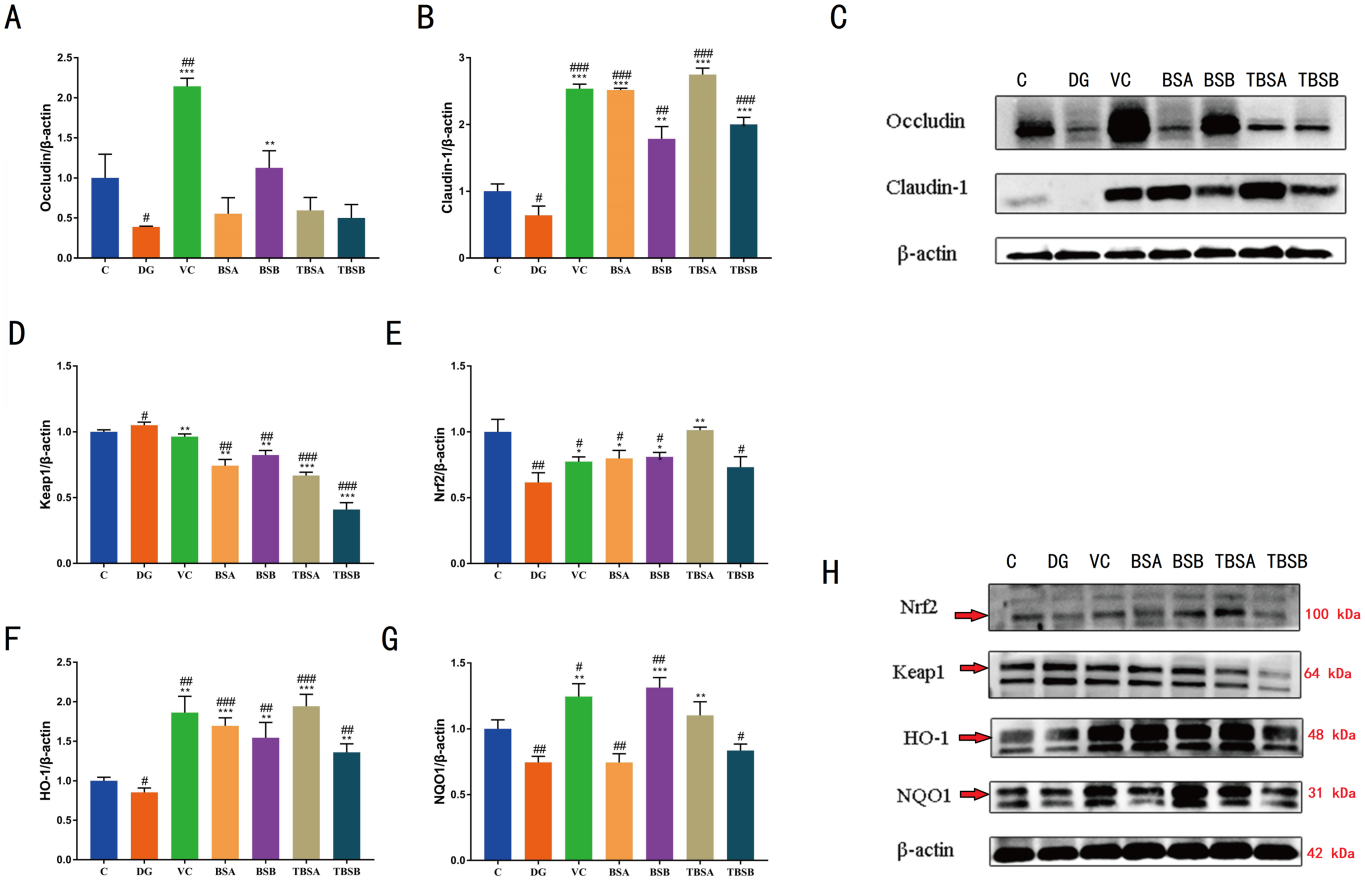
**(A–C)** Western blot analysis of occludin and claudin-1 proteins in various experimental groups. **(D–H)** Western blot analysis of key proteins in the Keap1/Nrf2 signaling pathway, including KEAP1, NRF2, HO-1, and NQO1. The symbol # indicates comparison with the C group, while * represents comparison with the D-galactose induced oxidative injury group (DG group).

A comparative analysis was conducted to evaluate the efficacy of *B. subtilis* (BS3 and BS7) in ameliorating D-galactose-induced impairment of gut barrier function in mice, with a focus on changes in tight junction protein (TJs). TJs, e.g., claudin-1 and occludin levels was significantly decreased in DG group compared with the C group (*p* < 0.05), while the opposite was true in groups BSA and BSB. Moreover, both the TBSA and TBSB groups showed a significant upregulation of claudin-1 protein level compared to the DG group (*p* < 0.05). Particularly, the TBSA group exhibited a markedly higher level of claudin-1 protein compared to the DG group (*p* < 0.001). No significant differences were observed between groups C and TBSA, nor between groups C and TBSB. These findings indicated that intervention with BS3 or BS7 effectively reversed the downregulation of tight junction proteins and mitigated intestinal barrier damage induced by D-galactose (Figures 4A–C).

### Effect of *Bacillus subtilis* on Keap-1/Nrf-2 pathway in D-galactose-exposed mice

The Keap-1/Nrf2 signaling pathway is essential for controlling oxidative stress in the body because it activates antioxidant response components. To further investigate the potential antioxidant mechanism of *B. subtilis*, we analyzed the protein expression of HO-1, NQO-1, NRF-2 and KEAP-1 in the liver of different groups of mice. In comparison to the C group, our findings demonstrated a considerable upregulation of KEAP-1 (*p* < 0.01) and a decrease in NRF-2 (*p* < 0.01) in the DG group. In contrast, intervention with BS3 and BS7 (TBSA group and TBSB group), led to a marked increase in NRF-2 (*p* < 0.01) and a significant reduction in KEAP-1 (*p* < 0.05) protein expression relative to the DG group. Notably, there was no significant difference in the relative levels of KEAP1 and NRF2 proteins between groups C and TBSA. To verify the activation of downstream targets of the Keap-1/Nrf-2 pathway, the protein levels of HO-1 and NQO-1 were further analyzed. The livers of mice exposed to D-galactose (the DG group) had significantly lower levels of HO-1 and NQO-l protein expression than the C group (*p* < 0.05). Treatment with BS3 or BS7 reversed this effect, as shown by higher levels of HO-l and NOQ-l proteins in the groups TBSA and TBSB than in the DG group (*p* < 0.05). The results indicated that *B. subtilis* including BS3 and BS7 intervention activates the hepatic Keap-1/Nrf-2 pathway to alleviate oxidative stress induced by D-galactose (Figures 4D–H).

### Acquisition of data and analysis of gut microbial diversity

**Figure 5:**
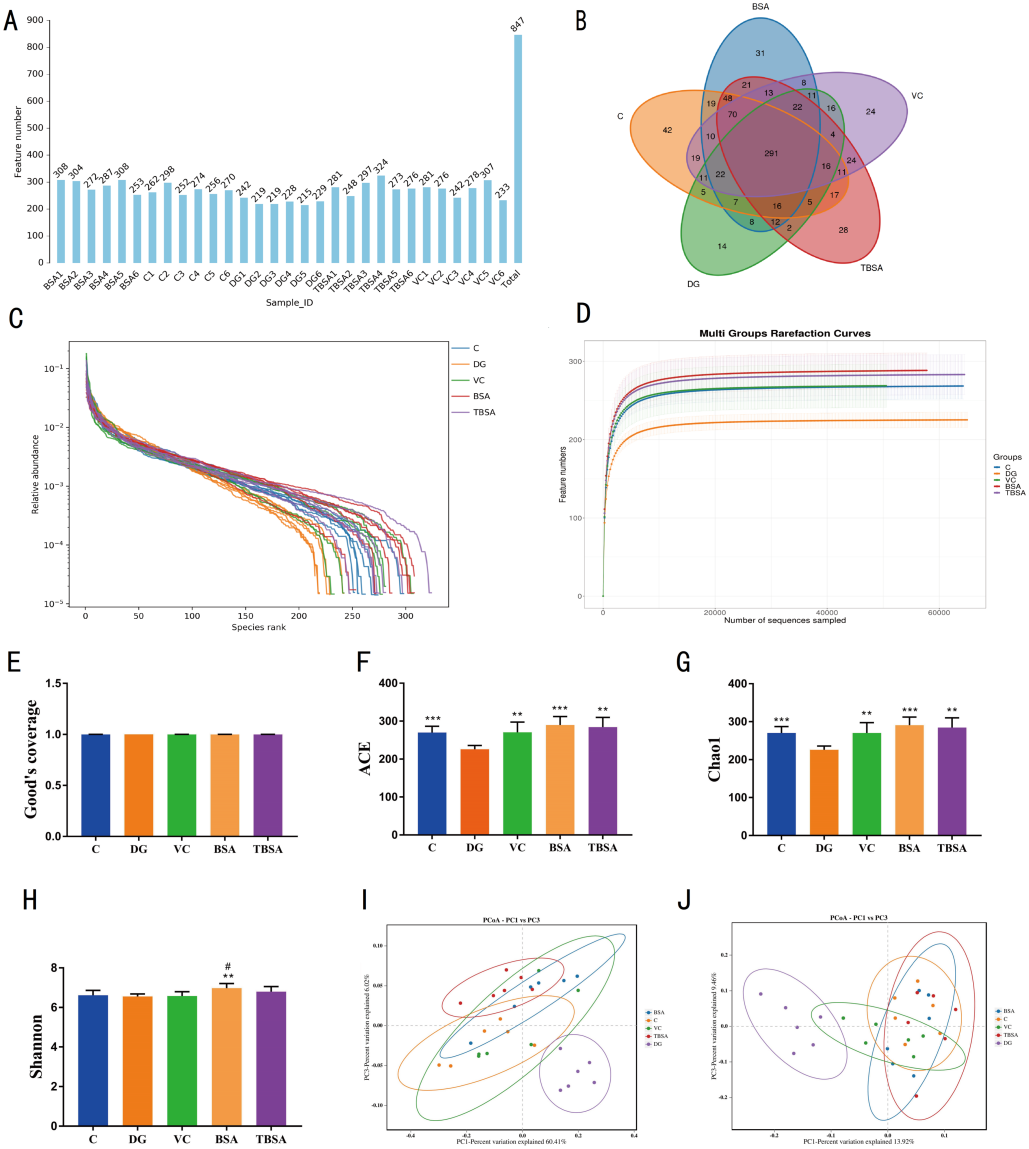
The impact of BS3 on alterations in microbial richness and composition in response to D-galactose exposure. **(A)** The OTUs count in each sample. **(B)** The Venn diagram displayed the distinct and overlapping operational taxonomic units (OTUs) among the five groups. **(C)** The rank abundance curve. **(D)** The rarefaction curve. **(E–H)** The alpha diversity analysis. **(I,J)** The PCoA analysis was conducted using the weight and unweighted UniFrac distance. The symbol # indicates comparison with the C group, while * represents comparison with the D-galactose induced oxidative injury group (DG group).

Based on previous findings indicating superior antioxidant properties of BS3 over BS7, we selected the BS3 strain for further investigation. Amplicon sequencing was performed on the colon contents of C, DG, VC, BSA and TBSA groups, and a total of 1,201,718 original sequences were obtained (C = 629,986, DG = 571,732, VC = 629,986, BSA = 571,732, TBSA = 629,986). Following quality control, 2,095,969 effective sequences were obtained from all samples (C = 629,986, DG = 571,732, VC = 629,986, BSA = 571,732, TBSA = 629,986). These eligible sequences were clustered into 847 OTUs and each sample contained between 215 and 308 OTUs (Figure 5A).

In addition, groups C, DG, VC, BSA and TBSA contained 42, 14, 24, 31 and 28 unique OTUs, respectively (Figure 5B). Moreover, the rarefaction and rank abundance curves were saturated and smooth, indicating the accuracy and completeness of sequencing results (Figures 5C,D).

The alpha diversity index was calculated according to the OTUs distribution to evaluate the changes in gut microbial diversity and richness. Compared to the C group, the DG group showed a noteworthy decrease in Chao1 (C: 270.1667 ± 6.9936, DG: 225.8333 ± 4.0387, *p* = 0.00058) and ACE indices (C: 269.8835 ± 6.7984, DG: 225.9089 ± 4.0888, *p* = 0.0005), while no significant difference was observed in Shannon index between the two groups (C: 6.6181 ± 0.0995, DG: 6.5564 ± 0.0516，*p* > 0.05) (Figures 5E–H), indicating that D-galactose significantly reduced gut microbial richness but had no impact on its diversity. However, BS3 and VC interventions could restore the reduction in gut microbial richness induced by D-galactose, as indicated by significantly higher Chao1 and ACE indices in the TBSA and VC groups compared to the DG group (*p* < 0.05 or *p* < 0.01), and it is worth emphasizing that the Chao1 and ACE indices in the TBSA and VC groups were not significantly different from those in the C group, suggesting that VC and BS3 interventions could alleviate the reduction in gut microbial richness induced by D-galactose (Figures 5E–H). To further explore the effect of BS3 on gut microbiota in D-galactose-exposed mice, beta diversity was analyzed, as shown in the PCoA plots (Figures 5I,J), samples from the C, BSA, VC and TBSA groups were clustered together and separated from the DG group, suggesting that D-galactose could lead to changes in the gut microbial composition, and the intervention of BS3 and VC effectively restore gut microbial profile and composition.

### The impact of *Bacillus subtilis* on the gut microbial composition of mice exposed to D-galactose

**Figure 6:**
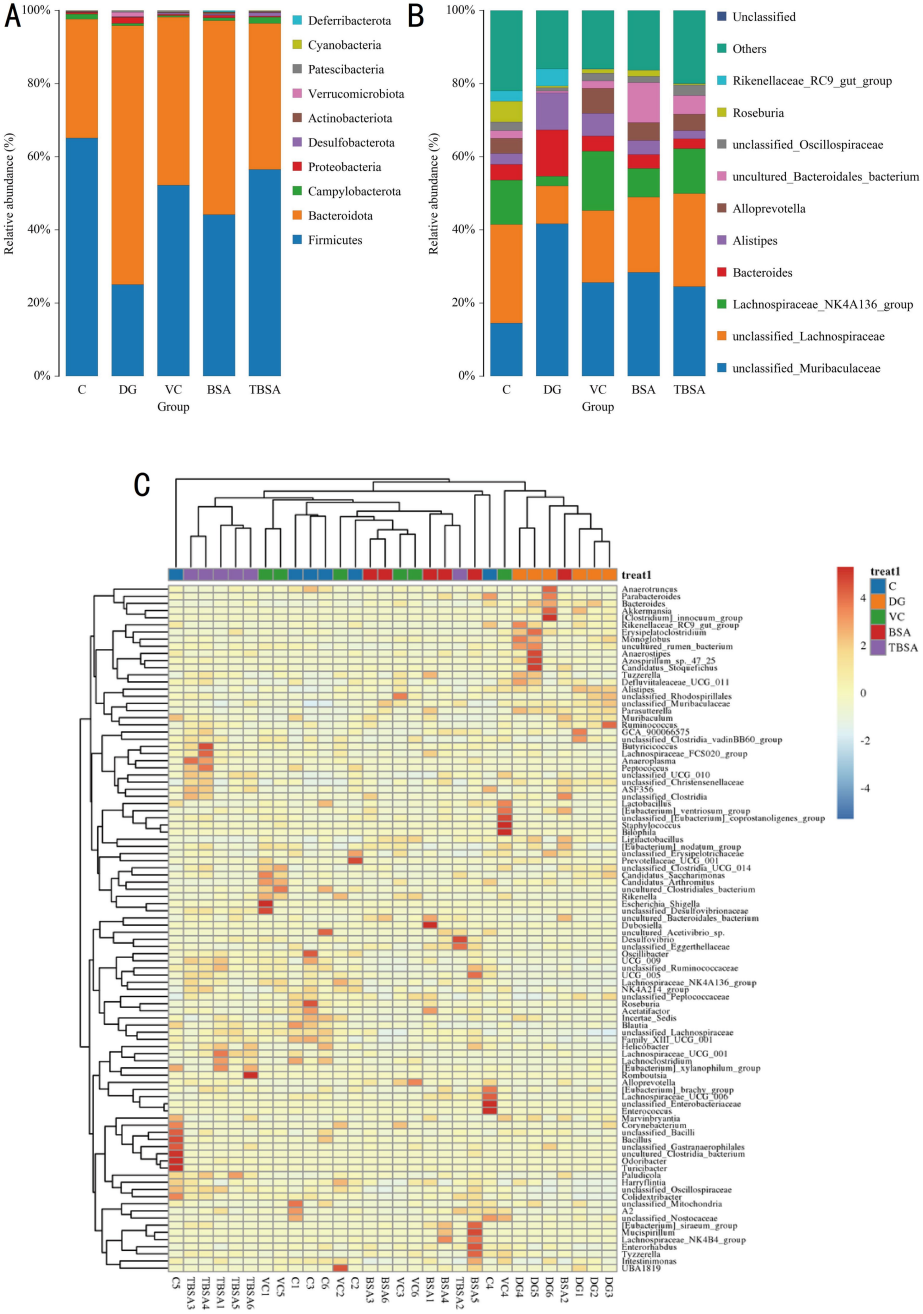
**(A,B)** The relative abundance of gut microbiota at the phylum and genus levels in five groups. **(C)** The heatmap displays the distribution of bacterial genera across five groups.

Thirteen phyla and one hundred and seventy nine genera were identified from 30 samples. Firmicutes (C: 65.14%, DG: 25.11%, VC: 52.56%, BSA: 44.17%, TBSA: 56.53%) and Bacteroidota (C: 32.49%, DG: 70.73%, VC: 45.96%, BSA: 53.02%, TBSA: 39.97%) were the dominant phyla in all groups, comprising over 95% of the bacterial composition. Other phyla such as Actinobacteriota (C: 0.26%, DG: 0.14%, VC: 0.20%, BSA: 0. 51%, TBSA: 0.23%), Verrucomicrobiota (C: 0.00073%, DG: 1.23%, VC: 0.017%, BSA: 0.0012%, TBSA: 0.013%), Patescibacteria (C: 0.12%, DG: 0.38%, VC: 0.38%, BSA: 0.092%, TBSA: 0. 27%), Cyanobacteria (C: 0.08%, DG: 0.05%, VC: 0.085%, BSA: 0.062%, TBSA: 0.092%) and Deferribacterota (C: 0.00%, DG: 0.00%, VC: 0.00%, BSA: 0.33%, TBSA: 0.00025%) were present in low abundance (Figure 6A). At the genus level, *unclassified_Muribaculaceae* (C: 14.51%, VC: 25.60%, BSA: 28.39%, TBSA: 24.51%) and *unclassified_Lachnospiraceae* (C: 26.96%, VC: 19.65%, BSA: 20.58%, TBSA: 25.44% %) were the predominant bacterial genera in C, VC, BSA and TBSA groups, whereas in the DG group, *unclassified_Muribaculaceae* (41.68%) was the most dominant genera followed by *Bacteroides* (12.73%) (Figures 6B,C).

**Figure 7:**
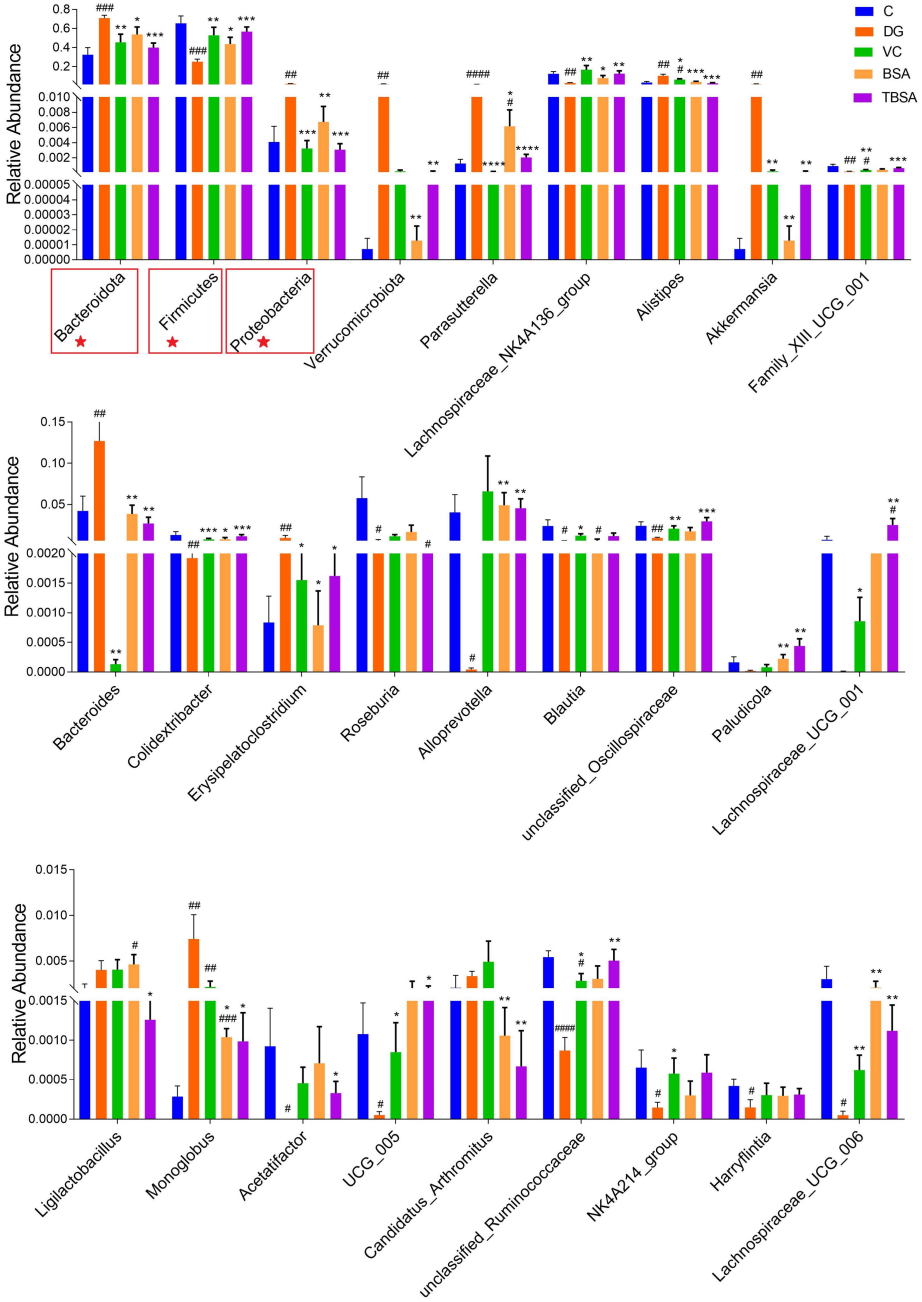
Statistical evaluation of differentially abundant bacterial taxa was conducted across five distinct groups, with specific focus on both the phylum (marked with ★) and genus levels. *p < 0.05 and **p < 0.01. The symbol # indicates comparison with the C group, while * represents comparison with the D-galactose induced oxidative injury group (DG group).

Metastats analysis was employed to identify differential bacteria at the phylum and genus levels. The DG group had significantly higher levels of Proteobacteria and Bacteroidota phyla, and lower levels of Firmicutes compared to the C group (*p* < 0.01). Simultaneously, it was calculated that the Firmicutes/Bacteroidota ratio was significantly decreased in the DG group compared to the C group. D-galactose resulted in a decrease in the Firmicutes/Bacteroidetes. At the genus level, significant differences were also observed in 15 bacterial genera between groups C and DG, of which six bacterial genera (*Parasutterella*, *Alistipes*, *Akkermansia*, *Bacteroides*, *Erysipelatoclostridium*, and *Monoglobus*) exhibited an obvious increase in the DG group compared to the C group (*p* < 0.05), and nine bacterial genera (*unclassified_Oscillospiraceae*, *Lachnospiraceae_NK4A136_group*, *Family_XIII_UCG_001*, *Colidextribacter*, *UCG_005*, *Harryflintia*, *Lachnospiraceae_UCG_006*, *Roseburia*, and *Acetatifactor*) was significantly decreased (*p* < 0.05). However, the BS3 intervention restored most of the above differential bacterial phyla and genera to group C levels. In addition, BS3 intervention could further optimise the gut microbial composition of mice exposed to D-galactose by increasing the abundance of *Alloprevotella*, *Paludicola* and *Lachnospiraceae_UCG_001* and decreasing the abundance of *Ligilactobacillus* (Figure 7).

**Figure 8:**
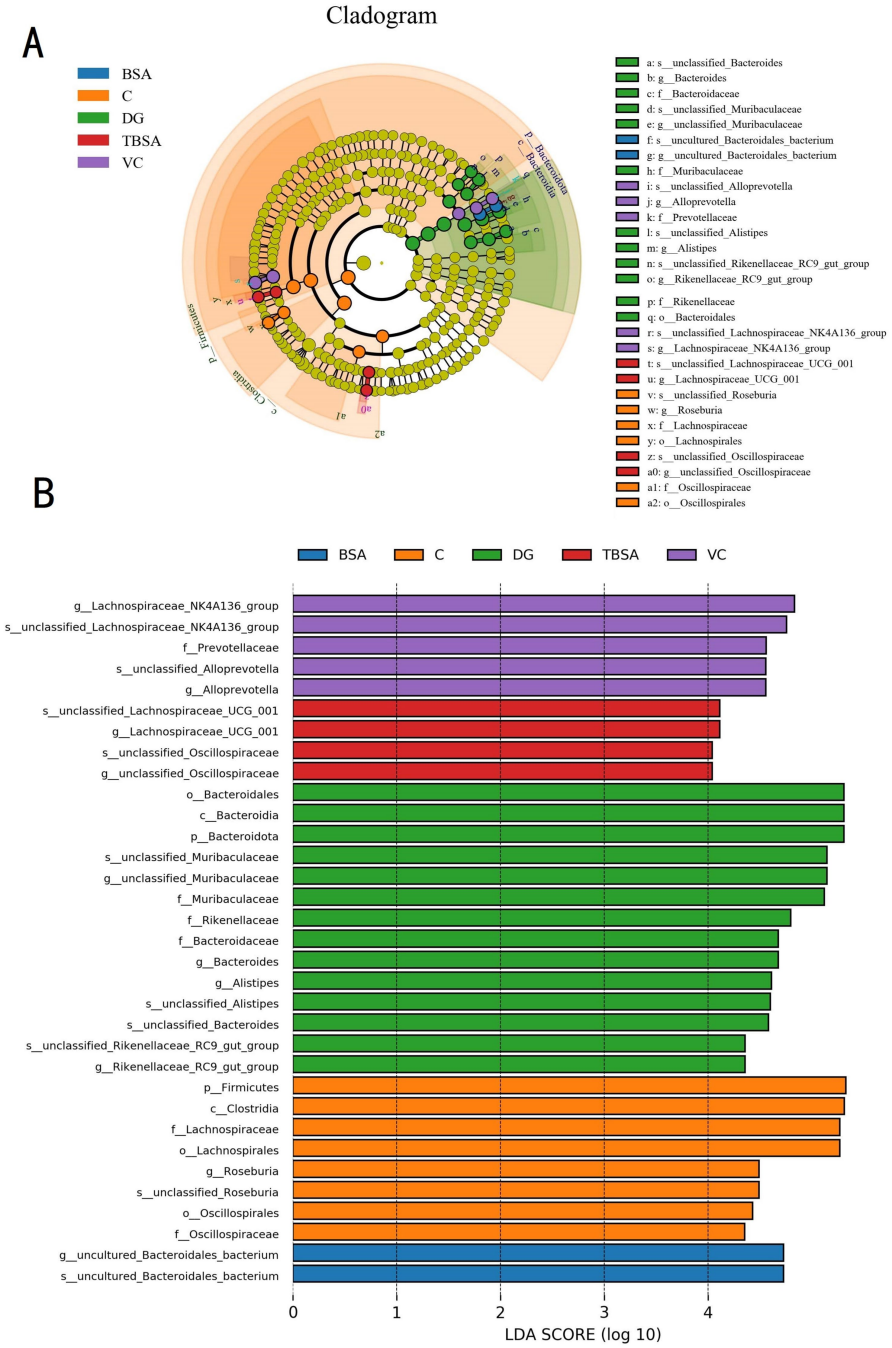
LEfSe analysis combined with LDA scores revealed the differential biomarkers among five groups. **(A)** The cladogram illustrates the phylogenetic distribution of diverse bacteria. **(B)** The linear discriminant analysis (LDA) scores, with thresholds exceeding 4, were indicated statistically significant differential bacteria.

Moreover, the LEfSe analysis and linear discriminant analysis (LDA) scores also showed similar results, with the DG group showing enrichment of the genera *Alistipes* and *Bacteroides*, whereas these were less abundant in the TBSA group. Additionally, the TBSA group showed enrichment of beneficial bacteria such as *Lachnospiraceae_UCG_001* and *Oscillospiraceae*, further highlighting differences in bacterial composition (Figure 8).

## Discussion

The detrimental impact of oxidative stress on both human and animal health has attained growing attention in scientific research. Evidence from studies indicates that antioxidants play a crucial role in counteracting oxidants, inhibiting lipid peroxidation, and halting free radical chain reactions before they inflict damage on vital biological molecules (Pereira et al., 2021; Pavlidis, 2022). Therefore, the intake of specific exogenous antioxidants is considered an effective strategy to alleviate oxidative stress and prevent diseases. However, certain synthetic antioxidants are discouraged due to concerns surrounding their thermal instability and potential toxicity (Wu and Venier, 2023). Therefore, there is an urgent need to explore natural non-toxic antioxidants that can reduce oxidative stress and prevent related diseases.

Probiotics, particularly those inhabiting the gastrointestinal tract, are promising sources of functional dietary antioxidants (Mishra et al., 2015). Although some probiotics (e.g., *Lactobacillus*) have been shown to ameliorate oxidative stress and liver injury (Ding et al., 2022), the antioxidant capacity of yak-isolated *B. subtilis* remained unexplored. Yaks are a species of high-altitude animals that are known for their ability to tolerate low oxygen levels and extreme cold, as well as for their strong antioxidant capacity (Wang Y. et al., 2022). Therefore, we systematically evaluated the effects of yak-isolated *B. subtilis* (BS3 and BS7) against oxidative stress and hepatic injury.

Serum biochemical indices in animals typically undergo dynamic fluctuations, which are influenced by dietary and environmental factors. These variations usually occur without significantly affecting the overall health of the host (Xu et al., 2021). However, disease states, oxidative stress, and environmental pollutants can induce alterations in these parameters, thereby serving as crucial indicators of host health status (Yang Z. et al., 2021). Increased levels of ALT and AST are reliable indicators of liver injury, as these enzymes are released from damaged hepatocytes into the bloodstream (Weber et al., 2019). Consistent with previous research (Li et al., 2018; Han et al., 2021), our findings demonstrated that D-galactose significantly increased AST and ALT indices in mice, suggesting hepatic injury. Histopathological examinations further corroborated the presence of hepatic injury in D-galactose-induced mice. Notably, intervention with *B. subtilis* was found to significantly reduce the elevated AST and ALT levels associated with D-galactose injection and to mitigate the observed pathological changes in liver tissue, suggesting that *B. subtilis* possess the potential to alleviate hepatic injury.

Previous studies have shown that ingested D-galactose can be metabolized into galactitol, a non-degradable compound. This accumulation of galactitol may trigger excessive production of oxidants, resulting in oxidative stress and subsequent tissue damage (Chen et al., 2022). Gao et al. reported that D-galactose administration in mice significantly reduced antioxidant capacity, accelerated ageing, liver injury and increased oxidative stress (Gao et al., 2021), which are in line with our finding. Our current study corroborates these findings by observing a significant decrease in antioxidant enzyme levels after D-galactose injection in mice (Gao et al., 2021). The antioxidant enzyme defense system is central to mitigating oxidative stress and associated pathologies by increasing the body’s resistance to oxidants (Luo et al., 2019). Among these enzymes, SOD exhibits multiple physiological functions, e.g., anti-aging, anti-inflammatory, and anti-tumor effects, and serves as the primary cellular defense mechanism against oxidative damage by neutralizing harmful metabolites and catalyzing superoxide anions (Tang et al., 2015). T-AOC serves as an integrative measure of an organism’s overall antioxidant potential, while GSH-Px and CAT are key enzymes involved in preventing oxidants accumulation and converting oxidants into less harmful substances (Zhao et al., 2016; Luo et al., 2019). MDA, a by-product of lipid peroxidation, serves as a key biomarker for the extent of oxidative stress. Elevated MDA levels can disrupt the structural integrity, permeability, and stability of biological membranes, ultimately compromising the overall health of the host (Pilarska et al., 2017; Queiroz et al., 2019).

Probiotics are recognized for their potent antioxidant properties, which confer protection against oxidative stress (Wang et al., 2023). Li et al. (2018) demonstrated that *Lactobacillus* could shield liver from oxidative stress by elevating T-AOC and SOD concentration. Similarly, in our finding, BS3 and BS7 increased SOD level in mice subjected to D-galactose-induced oxidative stress, suggesting a beneficial anti-oxidative effect of BS3 and BS7. Furthermore, Wu S. et al. (2022) indicated that *B. amyloliquefaciens* alleviated oxidative injury by upregulating antioxidant enzyme activities and modulating the Keap-1/Nrf2 signaling pathway, a critical regulatory mechanism governing the cytoprotective response to both endogenous and exogenous oxidative stress. This pathway plays a central role in regulating the oxidation-antioxidation system, which are essential for preserving organism’s overall antioxidant capacity and defensing oxidative injury (Long et al., 2022). Previous studies exhibited that certain *Lactobacillus* species can enhance hepatic antioxidant enzyme expression by modulating the Keap-1/Nrf2 pathway (Li et al., 2018). Consistently, our results indicate that BS3 and BS7 treatment significantly downregulated KEAP1 protein expression while upregulating NRF2 and HO-1 levels in D-galactose-induced mice. Thus, *B. subtilis* can improve hepatic antioxidant enzyme expression by regulating the Keap1/Nrf2 pathway, thereby mitigating D-galactose-induced oxidative damage.

Early studies demonstrated that preserving gut barrier integrity is crucial for the effective functioning of complex intestinal processes. Maintaining this barrier is essential for ensuring proper intestinal functions and overall gut health (An et al., 2022). The physical barrier, composed of tight junction proteins (TJs), plays a critical part in preventing the translocation of pathogens, endotoxins, and other deleterious substances from the lumen into the bloodstream (Jin et al., 2017). Key components of this barrier include claudin-1 and occludin, which serve as important markers for evaluating intestinal permeability and barrier function (Cuellar et al., 2017; Zhou et al., 2017; Chen et al., 2021). Previous research has shown that compromised integrity of gut barrier is closely associated with oxidative damage (Wang et al., 2020). Similarly, our study revealed an obvious reduction in TJs levels (i.e., claudin-1 and occludin) in mice exposed to D-galactose. Moreover, impaired gut barrier allows intestinal bacteria and their toxic metabolites to enter the liver via the portal veins, contributing to hepatotoxicity and progression of liver diseases (Cui et al., 2019). Consequently, preserving the integrity of gut barrier has been identified as a viable strategy for mitigating liver and gut disorders. Probiotics have been shown to increase the relative level of TJ proteins, thereby mitigating the translocation of gut pathogens (Cordeiro et al., 2021). Specifically, probiotics such as *Lactobacillus plantarum* and *Bacillus amyloliquefaciens* have demonstrated efficacy in alleviating liver disease by maintaining the integrity of gut barrier (Li et al., 2018; Ge et al., 2023). In this study, BS3 and BS7 intervention led to a significant upregulation of TJ protein expression, indicating their potential to restore the physical barrier and mucosal integrity of the intestine by repairing TJ proteins, thereby ameliorating oxidative injury and its associated damage.

The link between gut microbial composition and richness and host oxidative status has been established in previous studies (Li et al., 2018; Qin et al., 2022). This investigation showed that exposure to D-galactose led to gut injury and a decrease in gut microbial abundance, corroborating the findings reported by Li et al. (2018) and Sheng et al. (2022). Gut injury has been shown to negatively affect the survival of commensal microorganisms, resulting in microbial dysbiosis (Liao et al., 2022). A stable gut microbiota acts as a critical biological barrier, preventing the establishment and proliferation of pathogenic and opportunistic pathogens, thereby maintaining the integrity of gut barrier (Liu et al., 2019). However, disruption of the microbial balance impairs the gut barrier and immunity, increasing susceptibility to pathogen invasion (Gai et al., 2021). In addition, it has been shown that in the state of gut microbiota dysbiosis, certain bacterial species and their metabolites can translocate across the gut epithelial barrier and impair other organ systems, e.g., potentially exacerbating liver injury (Zheng and Wang, 2021). Our results demonstrated that BS3 effectively modulates the gut microbial composition and counteracts D-galactose-induced reduction in microbial richness.

We further analyzed the link between gut microbial community and oxidative injury. As shown in the results, the oxidative stress induced by D-galactose can lead to changes in certain bacteria that may play an essential role in the function of the gut. D-galactose led to a decrease in the Firmicutes/Bacteroidetes (F/B) ratio and an increase in the proportion of Proteobacteria. Notably, similar D-galactose-induced gut microbiota changes were also observed by Gao et al. (2021) and Sheng et al. (2022). In addition, Firmicutes were also shown to be significantly decreased in patients suffering from oxidative stress-related diseases (Sheng et al., 2022). Proteobacteria are the largest bacterial phylum. Some of its genera, such as *Escherichia coli* and *Salmonella*, are common pathogens or opportunistic causes of intestinal disease, which may lead to gastritis, diarrhea and even death (Li et al., 2019). Therefore, a higher abundance of Proteobacteria may increase the risk of pathogen infection. However, BS3 effectively reversed the alternation in gut microbiota in D-galactose-injected mice, with a decrease in Proteobacteria and an increase in Firmicutes. Moreover, BS3 intervention reversed D-galactose-induced decreases in the levels of *Alloprevotella*, *Oscillibacte*, *Lachnospiraceae_UCG_001*, and *Lachnospiraceae_NK4A136_group*, as well as increases in the levels of *Parasutterella*. Due to its ability to produce acetate and succinate, *Alloprevotella* has been shown to be strongly related to a reduced lifetime risk of cardiovascular disease (Xin et al., 2019). *Oscillibacte*, *Lachnospiraceae_UCG_001* and *Lachnospiraceae_NK4A136_group* are potential producers of SCFAs (Xie et al., 2016). Previous studies have shown that SCFAs are essential for the regulation of immunity, energy intake and intestinal metabolism (Xie et al., 2016; Liu et al., 2021). Meanwhile, SCFAs are central to positively regulating the gut microbiota and maintaining gut barrier function, both of which are critical for host health (Liu et al., 2021). Chiodini RJ demonstrated a significant association between *Paraspertella* and irritable bowel syndrome (Chiodini et al., 2015). Overall, BS3 has been shown to modulate the diminished and unbalanced gut microbiota by reducing the growth of harmful bacteria and promoting the growth of beneficial microorganisms, thus partially maintaining protective functions against D-galactose-induced oxidative stress and liver injury. The positive regulation effect of *B. subtilis* on gut microbiota and intestinal mucosal barrier may be one of its underlying mechanisms to alleviate oxidative stress.

## Conclusion

In conclusion, the study demonstrates that *B. subtilis* (particularly strain BS3) isolated from yaks, possesses therapeutic benefits against D-galactose-induced oxidative stress via regulation of the Keap1/Nrf2 signaling pathway. Additionally, BS3 has been shown to alleviated hepatic injury and gut microbial disorder, as evidenced by enhanced gut microbiota richness (*p* < 0.05) and a greater abundance of beneficial bacteria (*p* < 0.05), including members of Firmicutes phylum and *Oscillibacter* and *Lachnospiraceae_NK4A136* genera, while concurrently decreasing the abundance of potentially harmful bacteria, notably those within Proteobacteria phylum (*p* < 0.05), the positive regulation effect of BS3 on gut microbiota and intestinal mucosal barrier may be one of its underlying mechanisms to alleviate oxidative stress and hepatic injury. Despite these promising results, the current study acknowledges several limitations, including a relatively small sample size and the lack of a comprehensive analysis of metabolic changes. Therefore, further research involving both *in vitro* and *in vivo* models is needed to elucidate the metabolic mechanisms underlying the antioxidative effects of BS3.

## Data Availability

The original data in the research have been uploaded to the NCBI public database (BioProject number: PRJNA 1149669).
